# Left ventricular unloading and venting in veno‐arterial extracorporeal membrane oxygenation: the importance of cardiogenic shock aetiology in guiding treatment strategies

**DOI:** 10.1002/ehf2.14717

**Published:** 2024-02-08

**Authors:** Luca Baldetti, Guglielmo Gallone

**Affiliations:** ^1^ Cardiac Intensive Care Unit IRCCS San Raffaele Scientific Institute Milan Italy; ^2^ Division of Cardiology, Cardiovascular and Thoracic Department Città della Salute e della Scienza Turin Italy; ^3^ Department of Medical Sciences University of Turin Turin Italy

Veno‐arterial extracorporeal membrane oxygenation (VA‐ECMO) offers cardiorespiratory support to cardiogenic shock (CS) patients. However, VA‐ECMO, especially in the most common peripheral configuration, comes with the drawback of heightened afterload on the left ventricle (LV), primarily attributable to retrograde aortic flow that pressurizes the systemic arterial circulation. When the failing LV is unable to maintain vetriculo‐arterial coupling, a vicious circle ensues, characterized by worsening LV distention and LV end‐diastolic pressure (LVEDP) increase, reduced coronary perfusion and LV stroke volume, culminating into persistent aortic valve closure, LV stasis and thrombosis, pulmonary hypertension, arrhythmias, and overt pulmonary oedema.^1^ Institution of an additional circulatory support device can achieve LV decompression, alleviating the adverse effects of systemic pressure overload, and potentially increasing the likelihood of myocardial recovery and survival. While observational data suggest that LV decompression strategies may enhance outcomes, how to tailor LV decompression indication, modality and timing remains unclear.^1^, ^2^, ^3^, ^4^ This is particularly relevant as decompression strategies come at the biological cost of non‐negligible procedural and device‐related complications.^5^

The paper by Kang and colleagues published in this issue of *ESC Heart Failure* ^6^ presents important novel insights that may help to craft individualization of LV unloading/venting strategies in CS patients requiring VA‐ECMO support. This study is a retrospective single‐center cohort experience including consecutive patients who underwent VA‐ECMO for CS, analysing the interaction of CS aetiology with the association between LV decompression and 90‐day all‐cause mortality. Patients with post‐cardiotomy CS were excluded. Among 128 included patients, the aetiology of CS was AMI in 55.5% and non‐AMI in 44.5%. The latter included 73.8% of patients with acute decompensated HF‐related CS (ADHF‐CS), 17.1% with myocarditis and 6.8% with other aetiologies. LV unloading/venting was performed in 43.0% patients, of which intra‐aortic balloon pump (IABP) was used in 48 (87.3%) patients, atrial transseptal venting in three (5.5%) patients, and both in four (7.3%) patients. Of note, no other LV unloading/venting strategies were adopted, as microaxial flow pump devices (mAFP) are not currently approved in South Korea. As compared to AMI‐CS, non‐AMI‐CS patients were younger, less frequently experienced cardiac arrest, had lower critical illness scores, and lower LV ejection fraction. Importantly, non‐AMI CS patients were more frequently bridged to heart transplant (29.8% vs. 2.8%, *P* < 0.001) as compared to AMI‐CS patients. Patients undergoing LV unloading/venting were younger, less frequently experienced cardiac arrest prior to ECMO implant and underwent nominally higher rates of heart transplant as compared to patients with ECMO alone (22.8% vs. 9.6%, *P* = 0.078). Consistently with the high‐risk characteristics of this CS cohort, in‐study fatality was elevated, with 49.1% of non‐AMI CS and 56.3% of AMI‐CS patients experiencing 90‐day mortality. The Authors found a significant interaction in the association between LV unloading/venting and 90‐day mortality with CS aetiology (*P* for interaction = 0.029), so that LV unloading/venting was associated with reduced mortality among patients with non‐AMI‐CS (adjusted HR 0.37, 95% CI 0.14–0.96), but not among AMI‐CS patients (adjusted HR 1.96, 95% CI 0.90–4.27). While limited by the confounding inherent to the observational nature of the analysis, the study prompts us to consider the importance of CS aetiologies to guide LV unloading/venting indication and modality. Indeed, CS aetiologies are associated with differential clinical presentations that summarize peculiar underlying CS pathophysiological drivers and expected disease trajectories. AMI‐CS generally presents with small LV cavities, euvolaemia, no chronic neurohormonal adaptation and low tolerance to deranged haemodynamics and hypoperfusion. In this cohort, myocardial recovery is expected as the desired outcome and VA‐ECMO is primarily implanted as a bridge to recovery. Conversely, ADHF‐CS, the most frequent non‐AMI‐CS aetiology (and a growing clinical entity in contemporary practice), is characterized by dilated LVs with prominent afterload‐mismatch and impaired preload reserve, frequently associated with severe mitral regurgitation, biventricular dysfunction, peripheral congestion and hypervolemia. These patients are frequently affected by advanced chronic heart failure and are more tolerant to hypoperfusion thanks to chronic adaptation mechanisms. As an ADHF‐CS admission may herald the need of definitive heart replacement therapies, myocardial recovery is less frequently the expected outcome of this population, and mechanical circulatory supports are often primarily meant as bridge to LVAD/heart transplant (indeed, in the present study, rate of heart transplantation was 10‐fold higher in the non AMI‐CS cohort as compared to the AMI‐CS cohort). In this framework, the different clinical and haemodynamic treatment goals, and the different interactions in patient‐device haemodynamic effects are important considerations in guiding the type of LV unloading/venting strategy.

## Treatment goals of left ventricle decompression

**Figure 1:**
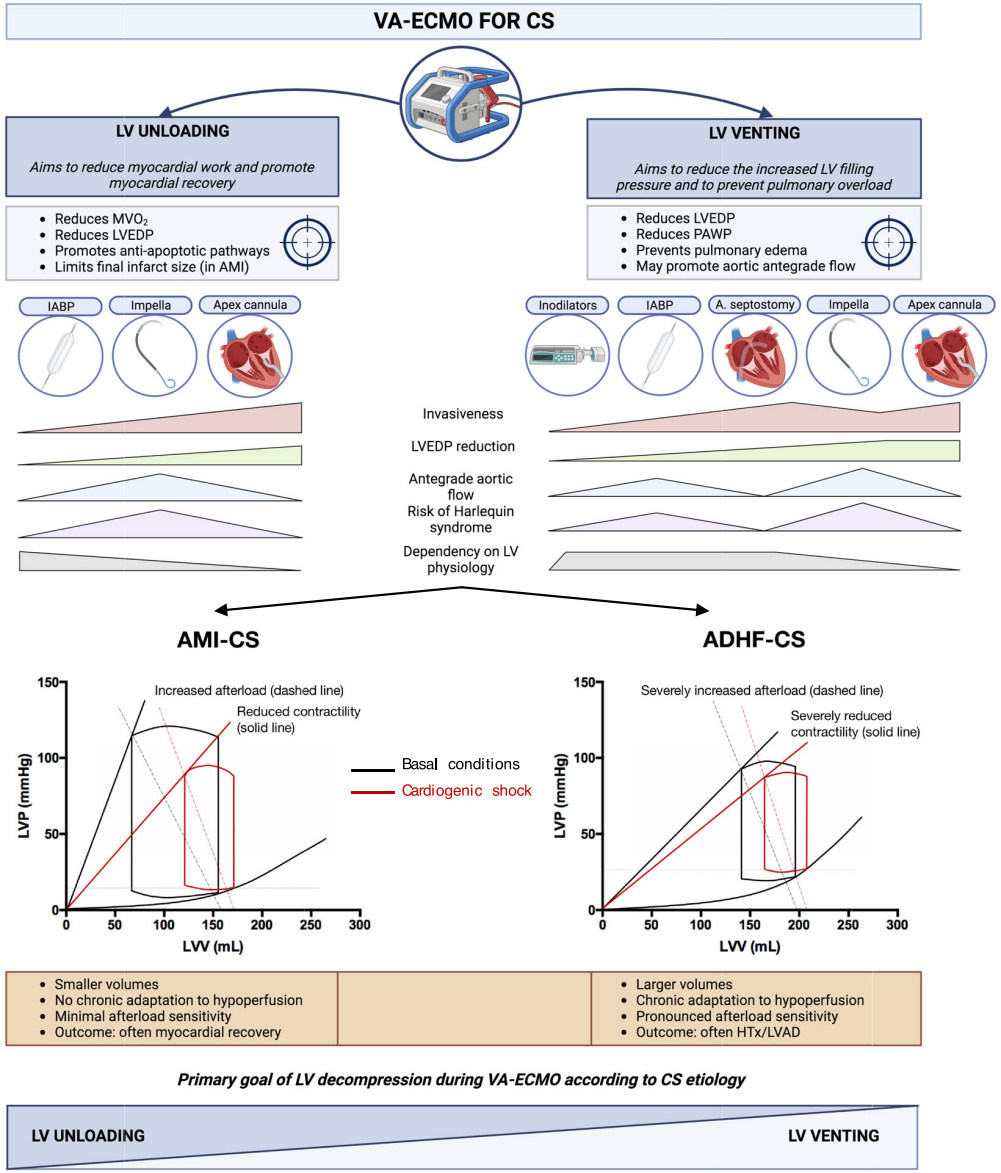
Summary of LV unloading and venting strategies aims, devices and appropriateness according to CS aetiology. ADHF‐CS, acute decompensated hear failure‐related cardiogenic shock; AMI‐CS, acute myocardial infarction‐related cardiogenic shock; CS, cardiogenic shock; HTx, heart transplant; IABP, intra‐aortic balloon pump; LV, left ventricle; LVAD, left ventricular assist device; LVEDP, left ventricular end‐diastolic pressure; LVP, left ventricular pressure; LVV, left ventricular volume; MVO_2_, myocardial oxygen consumption; PAWP, pulmonary artery wedge pressure.

LV decompression is carried to achieve two primary goals: LV venting and LV unloading. LV venting aims primarily to reduce pulmonary congestion among patients with increased LV filling pressures, to protect the lungs and to improve gas exchange. LV unloading primarily aims to reduce myocardial work to enhance myocardial recovery. While often interpreted as overlapping terms, conceptual differences with important clinical implications are, in fact, present (*Figure* *1*). LV unloading implies an effort to favour myocardial recovery maximizing myocardial work reduction achieving the lowest LVEDP and, possibly, also actively replacing LV work in producing cardiac output.^1^ Accordingly, IABP and mAFP are to be considered LV unloading strategies by concomitantly reducing LVEDP and enhancing cardiac output. These devices have also venting properties by reducing pulmonary congestion and preserving lung function. Conversely, atrial septostomy‐based modalities and inodilators are purely venting strategies, as they either chiefly achieve left atrial pressure reduction over LVEDP reduction, with associated reduction in cardiac output (septostomy), or reduce LVEDP and PAWP while increasing CO at the expense of an increased myocardial work (inodilators). In addition, select venting strategies may also prevent LV stasis, and prevent LV/aortic thrombosis. While both unloading and venting are desirable among VA‐ECMO supported CS patients, LV unloading is a pivotal target among patients with acute myocardial damage (i.e. AMI‐CS) to maximize the chances of myocardial recovery by promoting activation of anti‐apoptotic signalling pathways. Thus, left atrial septostomy might not be the desirable strategy in AMI‐CS potentially explaining the neutral results of the recent EARLY‐UNLOAD randomized trial comprising 66.4% of AMI‐CS patients.^7^

## Interaction of left ventricle decompression strategy with acute myocardial infarction‐related cardiogenic shock and acute decompensated heart failure‐related cardiogenic shock pathophysiology

Despite being an unloading device, the intensity of haemodynamic improvement achieved by IABP, is strongly dependent on residual LV pump function and LV afterload‐dependency. Accordingly, IABP shows the most prominent haemodynamic impact among VA‐ECMO supported patients with pre‐IABP pulse pressure >10 mmHg as opposed to those with a pulse pressure <10 mmHg.^8^ This is in line with prior knowledge regarding IABP patients not on VA‐ECMO support, and suggests that a IABP‐based unloading strategy may better suit the ADHF‐CS rather than AMI‐CS pathophysiology.^9^ While evidence supporting the benefit of IABP unloading in the setting of AMI‐CS patients on VA‐ECMO remains controversial, this pathophysiological reasoning may explain the neutral effect of LV decompression among AMI‐CS patients, mostly achieved by IABP, observed in the paper by Kang et al, and the concomitant three‐fold reduction in all‐cause mortality observed with LV decompression in non‐AMI‐CS patients. Conversely, mAFP devices might best suit the AMI‐CS physiology, by acting independently of residual pump function, and we are eagerly awaiting the results of studies addressing this hypothesis (NCT01633502; NCT05506449). Importantly, the considerations on the best patient‐device haemodynamic interactions must always be balanced against haemocompatibility‐related complications that appear to be more frequent in mAFP‐ versus IABP‐unloaded patients.^5^, ^10^

## Additional considerations

As CS emerges as a time‐sensitive disease, benefits of unloading/venting strategies may also be waned by delays in their institution. Retrospective data suggests that an ‘early’ venting strategy (spanning from before to few hours after VA‐ECMO cannulation) may be associated with lower odds of hospital mortality.^11^ Beyond these observations and depending on the degree of pulmonary compromise of the studied population, considerations related to the risk of Harlequin syndrome shall be included in future studies addressing decompressive strategies during VA‐ECMO. Indeed, devices that lower central aortic impedance (IABP) or directly increase LV‐to‐aorta flow (mAFP) may facilitate a greater amount of de‐oxygenated blood returning from the failing lungs to flow to the supra‐aortic trunks, whereas pre‐ventricular venting strategies (atrial septostomy) may shunt the de‐oxygenated pulmonary circulation venous return and thus prevent upper‐body hypoxemia. Interaction of VA‐ECMO, residual lung function, and unloading device thus requires strict haemodynamic monitoring.^12^

Finally, VA‐ECMO liberation is a key step in the mechanical circulatory support weaning process. However, some patients may still require some degree of mechanical support owing to residual severe cardiac dysfunction after VA‐ECMO interruption. In this perspective, a pre‐planned strategy comprising preferential adoption of axillary insertion unloading devices may allow prolonged mechanical support—while providing opportunity for deambulation—after VA‐ECMO removal.

In conclusion, the present study offers a unique opportunity to ponder the current knowledge on mechanical LV unloading/venting during VA‐ECMO and suggests that—also in this setting—an individualized approach privileging assessment of CS aetiology, of pre‐existing chronic haemodynamic adaptations, of the interaction of the heart‐lung unit and the final goal of the mechanical LV unloading/venting strategy might guide device selection and maximize the benefits expected from this therapy.

## Conflict of interest

None of the authors has relevant conflicts of interest to disclose.

## Funding

No funding was required for this manuscript.
